# Feasibility of p-Doped Molecular Crystals as Transparent Conductive Electrodes via Virtual Screening

**DOI:** 10.1021/acs.chemmater.2c00281

**Published:** 2022-04-25

**Authors:** Tahereh Nematiaram, Alessandro Troisi

**Affiliations:** Dept. of Chemistry and Materials Innovation Factory, University of Liverpool, Liverpool L69 7ZD, U.K.

## Abstract

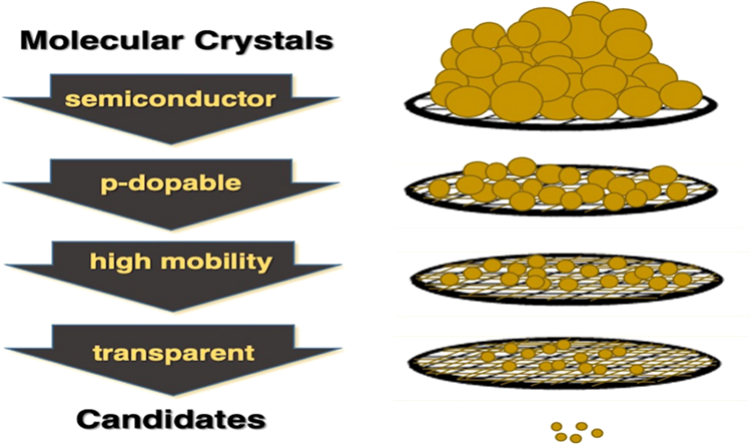

Transparent conducting materials are an essential component of optoelectronic devices. It is proven difficult, however, to develop high-performance materials that combine the often-incompatible properties of transparency and conductivity, especially for p-type-doped materials. In this work, we have employed a large set of molecular semiconductors extracted from the Cambridge Structural Database to evaluate the likelihood of transparent conducting material technology based on p-type-doped molecular crystals. Candidates are identified imposing the condition of high highest occupied molecular orbital (HOMO) energy level (for the material to be easily dopable), high charge carrier mobility (for the material to display large conductivity when doped), and a high threshold for energy absorption (for the material to absorb radiation only in the ultraviolet). The latest condition is found to be the most stringent criterion in a virtual screening protocol on a database composed of structures with sufficiently wide two-dimensional (2D) electronic bands. Calculation of excited-state energy is shown to be essential as the HOMO–lowest unoccupied molecular orbital (LUMO) gap cannot be reliably used to predict the transparency of this material class. Molecular semiconductors with desirable mobility are transparent because they display either forbidden electronic transition(s) to the lower excited states or small exchange energy between the frontier orbitals. Both features are difficult to design but can be found in a good number of compounds through virtual screening.

## 1 Introduction

Transparent conducting materials (TCMs) are of great practical interest due to their critical importance in information and energy-related technologies.^1,2^ The discovery of novel TCMs is notoriously challenging as potential materials should display simultaneously high electrical conductivity (which necessitates high mobility and/or high carrier concentration) and optical transparency, two incompatible properties that are rarely found in the same material. The goal, therefore, has been to find materials either exhibiting high conductivity despite a large band gap or being transparent despite a small band gap. Traditionally, large band gap materials constitute the main strategy for developing TCMs, where often an oxide with a band gap of larger than ∼3.2 eV, which favors visible spectrum transparency, is doped for higher concentration of charge carriers, either holes (p-type) or electrons (n-type), to achieve high conductivity.^3,4^ Indium oxide (In_2_O_3_) and tin dioxide (SnO_2_) often doped, respectively, with tin and fluorine are the most widely used TCMs.^5−7^ Despite their great advantages, the application of these compounds is unsustainable in the long term mainly due to the shortage of supplies and their brittle nature.^8,9^ Therefore, in recent years, a variety of new oxide as well as nonoxide TCMs have been emerged such as Ga_2_O_3_,^10^ BaSnO_3_,^11^ conducting polymers,^12^ carbon nanotubes,^13^ metallic nanowires,^14^ and graphene.^15^

**Figure 1:**
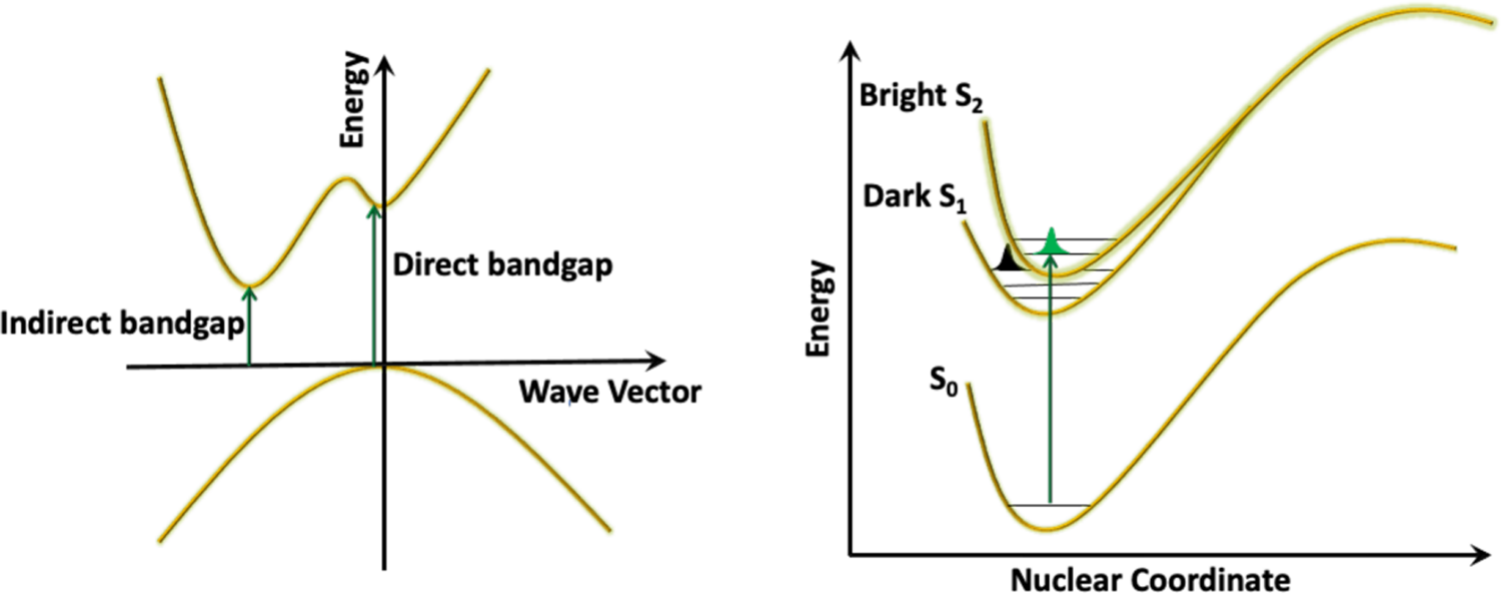
Two realizations of small band gap transparent materials. (Left) Band structure of a material with an indirect band gap, and (Right) the energy diagram of a molecule with a dark first excited state (S_1_) and a bright second excited state (S_2_). S_0_ indicates the ground state.

The observation that certain materials exhibit a significant disparity between optical and electronic band gaps has led to the development of transparent conductive materials with small electronic band gap and large optical band gap. Indirect band gap materials are the main known examples: the interband transitions at the electronic gap on absorption are of negligible intensity that requires the coupling with phonon modes (see Figure 1).^16^ These structures are especially relevant in thin-film-based applications as an indirect gap lower than the 3.2 eV threshold can be acceptable. This is the main mechanism in TCMs based on SnO as well as zinc blende boron phosphide,^16−18^ and it has been exploited to search novel TCMs via high-throughput screening.^19^

The majority of TCMs based on indirect gap materials employ n-type-doped semiconductors, including those commonly used in commercial devices.^2,20^ This is while, in many optoelectronic applications, ranging from interlayer electrodes in tandem cells to p–n heterojunctions and hole collection electrodes in photovoltaics and solar water splitting, high-performance p-type TCMs are required.^21−23^ Although some p-type materials have been proposed, for instance, Cu-based delafossite oxides, e.g., CuAlO_2_,^24^ and CuBO_2_,^25^ these structures remarkably lag in both their performance and device integration. There are two main reasons for the paucity of efficient p-type transparent oxides: first, oxide valence bands have low energy; thus, holes are exposed to compensation by defects such as oxygen vacancy and hydrogen leading to low p-type dopability.^26^ Second, the valence band maximum is primarily made up of localized oxygen 2p orbitals, resulting in a high (low) hole effective mass (mobility).^26,27^ As a result, the question still remains open as to whether there are promising transparent materials with high hole conductivity and what design rules could be exploited to design such materials.

Small organic molecules are a class of materials that has not received much attention in TCM-related research and technology. This is despite the fact that printed electronics based on small molecules, with conductive inks being the widely known representative example,^28,29^ have made significant advances indicating that TCM technology based on small molecules could be the key to producing low-cost, flexible devices. Molecular semiconductors, in contrast to oxides, show great promise for high conductivities.^30^ The current research has focused on improving their charge-transport characteristics, and as a result, they are projected to exhibit hole mobilities as large as 70 cm^2^/(V s).^31^ It has to be noted, however, that the mechanism of charge transport in molecular semiconductors is quite different than that of oxides. In contrast to oxides and, in general, to inorganic semiconductors, where charge carriers are fully delocalized and band model and resulting effective mass can successfully describe the charge transport, charge carriers in organic semiconductors are localized at one or several molecules due to the strong electron–phonon interactions.^32−34^ In low-mobility organic semiconductors, the charge carrier localizes at a single molecule resulting in the formation of a small-polaron and charge hopping models^35^ are often utilized to describe the charge transport. However, in the high-mobility molecular semiconductors of interest to this work, the charge carrier is delocalized over several molecules,^36,37^ and as a result, neither the band model nor the hopping model can describe the charge transport in this materials class. A number of advanced theories have been developed over the years to tackle the problem of charge transport in molecular semiconductors with several overviews of the area available in refs (32, 38), and (39).

Furthermore, a wide range of methods including small molecules, polyelectrolytes, covalent solids, Brønsted and Lewis acids, and elemental species are developed for their p-type doping,^40,41^ such that common organic semiconductors appear to be dopable with a range of different approaches.^42−44^ It is, however, important to note that doping an organic crystal without adversely affecting the crystalline quality is a challenging task due to the presence of weak intermolecular interactions and lattice mismatches. However, there has been outstanding progress in recent years that has been able to address these issues to a great extent, and practical strategies have been proposed for both p- and n-type doping with numerous articles and reviews of the area.^45−49^ For instance, exposing the pentacene films to iodine vapor is shown to be among the practical methods that leads to high mobility and conductivity, implying that the crystal structure remains undisturbed even at higher doping concentrations.^50^ The X-ray diffraction (XRD) evaluation of iodine doped pentacene has shown that iodine gets essentially intercalated between the planes of pentacene, meaning that the in-plane herringbone structure remains intact.^51^ As a result, doping does not deteriorate charge mobility and lead to high conductivities. Doping with iodine, therefore, seems a promising route for many organic semiconductors with a high-mobility plane in herringbone structures. Molecular dopants have also shown the ability to increase the conductivity of hole transport materials.^52−54^ The microscopic structure assessments of F_4_-TCNQ-doped pentacene films, with a doping concentration of ∼6%, by scanning tunneling microscopy have revealed that the dopant molecules diffuse into vacancies of the host lattice and do not degrade the crystalline structure^55^ and a similar mechanism is also in action in the F_4_-TCNQ-doped C8-BTBT films.^56^

It has to be noted that although the specific small dopants such as iodine and F_4_-TCNQ do not adversely affect the crystal packing, they often suffer from the fact that they are not considered as strong dopants and are volatile. Therefore, materials doped with such dopants are often de-doped, for example, when exposed to vacuum as discussed in, e.g., refs (57) and (58). Advances in vapor deposition techniques^59^ have also proven able to dope relatively large concentrations of molecules in lattices of molecular semiconductors while retaining the original crystal structure, such that the successful growth of pentacene and tetracene-doped *trans*-1,4-distyrylbenzene crystals with a doping concentration of up to 10% is reported.^60^

It should be emphasized, however, that the reason for transparency in molecular semiconductors differs from that of inorganic materials. Excitation in molecular semiconductors does not generate separated electron–hole pairs (Wannier–Mott exciton) and, therefore, the excitations are not described as the transition of electrons between bands. Due to the low dielectric constant and their narrow-band nature, the transition is, to the first order, localized on individual molecules, and the crystal absorption is determined by collective single-molecule excitations (Frenkel excitons).^61−64^ The energy of the lowest allowed transition in a molecule can be very different from the energy difference between the highest occupied molecular orbital (HOMO) and the lowest unoccupied molecular orbital (LUMO) known as HOMO–LUMO gap *E*_g_, and the latter is not a reliable predictor of the transparency of the material. For example, the excitation energy for the simplest excited states involving primarily the transition between HOMO and LUMO is reduced by the exchange energy between these two orbitals (which varies widely across molecules) in post-Hartree–Fock theories.^65^ The quantity *E*_g_ – *E*_S_, with *E*_S_ being the energy of the lowest allowed transition, is often referred to as the exciton binding energy,^66^ originating from the electrostatic interaction between electron and hole in the excited state that stabilizes this state relative to separate electron and hole states.^67^ Molecular semiconductors with *E*_g_ larger than the range of visible spectrum become transparent if the energy difference *E*_g_ – *E*_S_ is small enough to maintain the energy of the lowest allowed transition above the visible spectrum as well. Molecular semiconductors with a small *E*_g_ can also be transparent if the lowest excitation(s) of the molecule (i.e., with some of them presumably being in the gap) are forbidden. Figure 1 displays two realizations of small band gap transparent materials—the band structure of materials with an indirect band gap is depicted in the left panel, and the energy levels of a molecule with a dark first excited state and bright second excited state are depicted in the right panel.

Developing transparent molecular semiconductors is challenging because there are no intuitive rules to determine the oscillator strength of molecular electronic transitions or predict the exchange contribution to the lowest energy transition (except for the simpler case of donor–acceptor molecules^68^). When considering the combination of transparency and conductivity for TCMs, this becomes even more difficult as there is also a limited number of materials with desirable charge-transport properties. Therefore, the feasibility of TCM technology based on p-type-doped molecular crystals is difficult to explore by trial and error. It is also critical to note that for a material possessing appropriate conductivity and work function, either initially or through surface modification, it might be expected that an electron-transporting electrode can be used for hole injection/collection, as is the case for MoO_3–*x*_ and ITO.^69−71^ Accordingly, one might possibly consider organic materials based on the same principle, i.e., based on, for instance, materials with high electron affinities, e.g., F_4_-TCNQ commonly used as p-dopants, with a certain level of n-doping. However, it will be highly challenging to design organic n-doped hole-injection electrodes based on such an approach because a very few molecules might have large enough electron affinity as well as reasonable transparency. This further highlights the importance of p-dopable materials for hole-injection/collection electrodes.

In this paper, we employ a high-throughput virtual screening approach^19,72−75^ to search for transparent molecular semiconductors that also display desirable charge-transport properties. Starting from the extremely large database of >1 million crystal structures deposited in the Cambridge Structural Database (CSD),^76^ we identify a small number of potentially high-performance materials that could be exploited in respective optoelectronic applications. It is also important to note that considering the development of organic TCMs should not be seen as a competition against inorganic ones with an eventual winner. Organic TCMs may be, for example, the preferred choice for all-organic devices, where flexibility and low energy solution processing are essential requirements.

## 2 Methods and Computational Details

This work combines the results of several recent high-throughput studies developed within our group: (i) the screening of low HOMO–LUMO gap *E*_g_ molecules for interesting optoelectronic properties like singlet fission^77^ and temperature-activated delayed fluorescence^78^ and (ii) the screening of molecular semiconductors for high charge mobility^31^ and structural features that reduce the dynamic disorder.^79^ The key aspects of these data sets are summarized here for convenience. It is notable that although there are a number of excellent crystalline organic materials repositories, e.g., the Organic Materials Database,^80^ Organic Crystals in Electronic and Light-Oriented Technologies Database,^81,82^ and Atomic Structures and Orbital Energies of Crystal-Forming Organic Molecules,^83^ the work we present here cannot be done with any of these existing tools as the key point is to have a combined repository of excited-state properties and charge mobility.

### 2.1 Database

Our initial database is a set of molecular semiconductors extracted from over a million structures deposited in the Cambridge Structural Database (CSD),^76^ as detailed in ref (77). The considered structures are all organic (i.e., are composed of elements H, B, C, N, O, F, Si, P, S, Cl, As, Se, Br, and I) and cocrystals, polymers, disordered solids, duplicate structures, and materials containing molecules with more than 100 non-hydrogen atoms are excluded. In the first instance, this criteria led to a set of 250,000 molecules, all extracted using CSD Python API,^84^ and we employed single-point semiempirical and a range of density functional theory (DFT) calculations to compute the gap between HOMO and LUMO on a representative set of molecules using their X-ray geometries.^77^ As shown in the Supporting Information (SI) and will be discussed later in below; key characteristics are retained after geometry optimization. We obtained calibration curves to estimate high-level computed HOMO and LUMO energies (B3LYP/6-31G*) from the intermediate one (B3LYP/3-21G*). These calibrated orbital energies are used in the rest of the work when comparing orbital energy levels. We made a prescreening based on *E*_g_ to reduce the number of candidates from 250,000 to 40,000, enabling the calculation of both excited states and mobility on a set of molecular semiconductors with a computed molecular HOMO–LUMO gap in the range of [2, 4] eV. These molecules constitute the initial database of the present study, and the frontier orbital energy levels contribute to the identification of TCMs.

### 2.2 Excited-State Energy Calculations

For the isolated molecules contained in this database, the time-dependent density functional theory (TDDFT) calculations of the excited-state energies in their X-ray geometries at the M06-2X/def2-SVP level of the theory^85^ have been performed in ref (77). In the same reference, the computed singlet excited-state energies have been benchmarked against 100 experimental transition energy and a linear calibration curve was derived between calibrated *E*_S1_^c^ and computed *E*_S1_ values of singlet energy as *E*_S1_^c^ = 0.7502 × *E*_S1_ + 0.3269 with squared correlation coefficient *R*^2^ = 0.91. In this work, we use the lowest three singlet excited states obtained from that data set, calibrated with the same coefficients, and the corresponding oscillator strength to evaluate the lack of absorption in the visible range of the molecules in the data set. It is important to note that TDDFT (in the standard version which is used throughout this work) describes well excited states composed of multiple single excitations, but it is unable to describe states with double excitations like the A_g_ states in polyenes or the multiexcitonic states in singlet fission materials. The states that TDDFT misses are invariably dark, and this limitation will have no impact on the findings of this paper.

### 2.3 Mobility Calculations

The crystalline structure of the identified 40,000 molecular semiconductors, with computed HOMO–LUMO gap *E*_g_ of their X-ray geometries in the range of [2, 4] eV, was employed to compute approximated electron–phonon Hamiltonian parameters and then hole mobility from such parameters.^31^ In ref (31), transfer integrals between HOMO orbitals of two neighboring molecules are computed for the entire database. To each transfer integral, a vector *R* connecting the mass center of the molecules involved in the coupling is assigned. As such, we denote by *J*_1_ the largest transfer integral in absolute value and by *J*_2_, the second-largest transfer integral in absolute value whose associated *R*_2_ is nonparallel to *R*_1_ (i.e., *J*_1_ and *J*_2_ are predominant transfer integrals in different directions). By excluding materials with an overall too narrow bandwidth, i.e., |*J*_1_| < 0.1 eV, as well as those whose transport is predominantly one dimensional, i.e., |*J*_2_|/|*J*_1_| < 0.05, the set of remaining 4801 crystals, which are not necessarily high-mobility structures but a set that excludes those that will be definitely low mobility,^86^ are considered for further investigations. This approach is based on the observation that one-dimensional transport is not too efficient,^86^ but it also excludes crystals with pairs of molecules interacting strongly among them and weakly with the neighbors. The local electron–phonon coupling is computed using the normal mode projection method proposed in ref (87). One of the important aspects of the study was the developed novel multiscale quantum mechanical/molecular mechanics method for crystal’s phonon calculations, employing Gaussian ONIOM scheme,^88^ which has enabled calculations on such a large database, while exact methods are limited to a handful of structures.^89,90^ Finally, mobility was computed using transient localization theory (TLT),^39,91^ which is among the advanced theories that have been developed to illustrate the charge transport in molecular semiconductors. The charge mobility calculations in the framework of TLT are straightforward, enabling it to be an excellent model for high-throughput calculations.^31,86^ Furthermore, TLT has proved the ability to correctly predict the charge mobility in single-crystal thin-film transistors. As such, so far, 13 different high-mobility molecular semiconductors, including some of those reported in Table 1, have been evaluated in refs (86) and (90), and the absolute values of calculated mobilities are shown to be within ∼35% of experimental data. The comparison with experiments is particularly satisfactory when a series of related molecules characterized by the same group are considered, such as pentacene derivatives^86^ or BTBT derivatives,^90^ and the method was also shown to yield a predictive map of molecular semiconductors in ref (86).

**Table 1:** Frontier Orbitals Energy Levels (HOMO *E*_h_ and LUMO *E*_l_), the HOMO–LUMO Gap (*E*_g_), and the Experimentally Measured Hole Mobilities (μ) for a Subset of Structures Gathered from the Literature

material	*E*_h_ (eV)	*E*_l_ (eV)	*E*_g_ (eV)	μ (cm^2^/Vs)	refs
rubrene	–4.9	–2.3	2.6	7.1	(100)
C6-DBTDT	–5.6	–1.7	3.9	5.6	(101)
DNTT	–5.2	–2.2	3.0	3.0	(102)
pentacene	–4.6	–2.4	2.2	2.1	(103)
TES-ADT	–5.4	–3.1	2.3	1.8	(104)
NDT1	–5.1	–1.6	3.5	1.5	(105)
TIPS-PEN	–4.9	–3.0	1.9	1.2	(106)
DBA-IFD	–4.8	–2.9	1.9	1.0	(107)
hexacene	–5.0	–3.6	1.4	0.9	(108)
TIPS-ADT	–5.0	–2.7	2.3	0.6	(106)
pentaceno[2,3-*b*]thiophene	–5.0	–3.2	1.8	0.6	(109)

## 3 Results and Discussions

Potential TCM materials require strong conductivity and visible spectrum transparency. To maximize conductivity, one would logically strive to maximize both carrier concentration and charge carrier mobility (μ). In the most basic understanding of p-type doping, an integer number of electrons is transferred from the host to the dopant. To make this transfer efficient, the dopant’s electron affinity must be greater than the host’s ionization potential necessitating host materials with high-lying HOMO energy levels (*E*_h_).^41^ It is important to note that, due to scattering mechanisms such as ionized impurity scattering,^92^ carrier–carrier scattering,^93^ and electron–phonon scattering,^94^ adding too many charge carriers in the doping process can limit the overall mobility. Charge carrier concentration can also affect the transparency via the plasmon frequency relationship, which describes the oscillation of charge carriers within an applied field.^95^ Accordingly, if the amount of carrier concentration is large enough, the plasmon frequency can be shifted from the near-infrared range to the visible spectrum, to the detriment of transparency in the visible range. A viable strategy to overcome this bottleneck, therefore, would be to prioritize high mobility over high charge carrier concentration as also noted in a recent study.^96^ It has to be noted that in low-mobility materials where the charge transport is dominated by small polarons and is described by the hopping model, the carriers are trapped and cannot oscillate collectively. In these materials, small polarons, which can be excited to either a delocalized state or to a small-polaron state at a neighboring molecule, lead to optical transitions.^97^ It is demonstrated that, in these materials, the large number of doped carriers do not alter the plasmon frequency considerably and larger conductivity, which is accompanied by visible spectrum transparency can be obtained despite small mobilities. Relying on such strategy, recently new inorganic TCMs possessing small mobilities have been proposed.^98^ This mechanism, however, does not apply to molecular semiconductors because, on the one hand, as explained above, the charge carrier is delocalized over several molecules and not in a single molecule to result in small polarons, and on the other hand, inorganic materials can sustain greater doping without altering the crystal structure, so, one can have more carriers which compensate the lower mobility—a strategy not available for organic materials where crystallinity is important and one cannot dope heavily.^46^ Accordingly, the focus of our analyses is on the database of 4801 molecular semiconductors with computed mobilities, as explained above.

To measure transparency, we considered the energy of *E*_S_, where we have considered allowed the transitions with computed oscillator strength larger than 0.0005 (in the Supporting Information, we show how this threshold is expected to ensure good transparency for films of typical thickness). We have neglected the absorption of carriers, assuming that their absorption coefficient is comparable to that of the semiconductor above the threshold of the first absorption and considering a low doping concentration ∼2%. The transmittance in this situation for a typical film with a 500 nm thickness would be ∼95%, well above the threshold for considering a material transparent. In the SI, using the excited-state properties of oxidized molecules, these arguments are further detailed. This consideration is also often implicit in the majority of works on inorganic TCMs where the same objection applies. The material is transparent if *E*_S_ is larger than 3.26 eV, which is the boundary of ultraviolet–visible spectrum.^99^ It has to be noted that having computed the excited-state energies, *E*_g_ could also be removed from the analysis of this work. It will be, however, retained in the analysis (i) to establish whether screenings based on only orbital energy retain the same value and (ii) because, as discussed below, the energy difference between *E*_S_ and *E*_g_ offers insights into some materials’ transparency.

**Figure 2:**
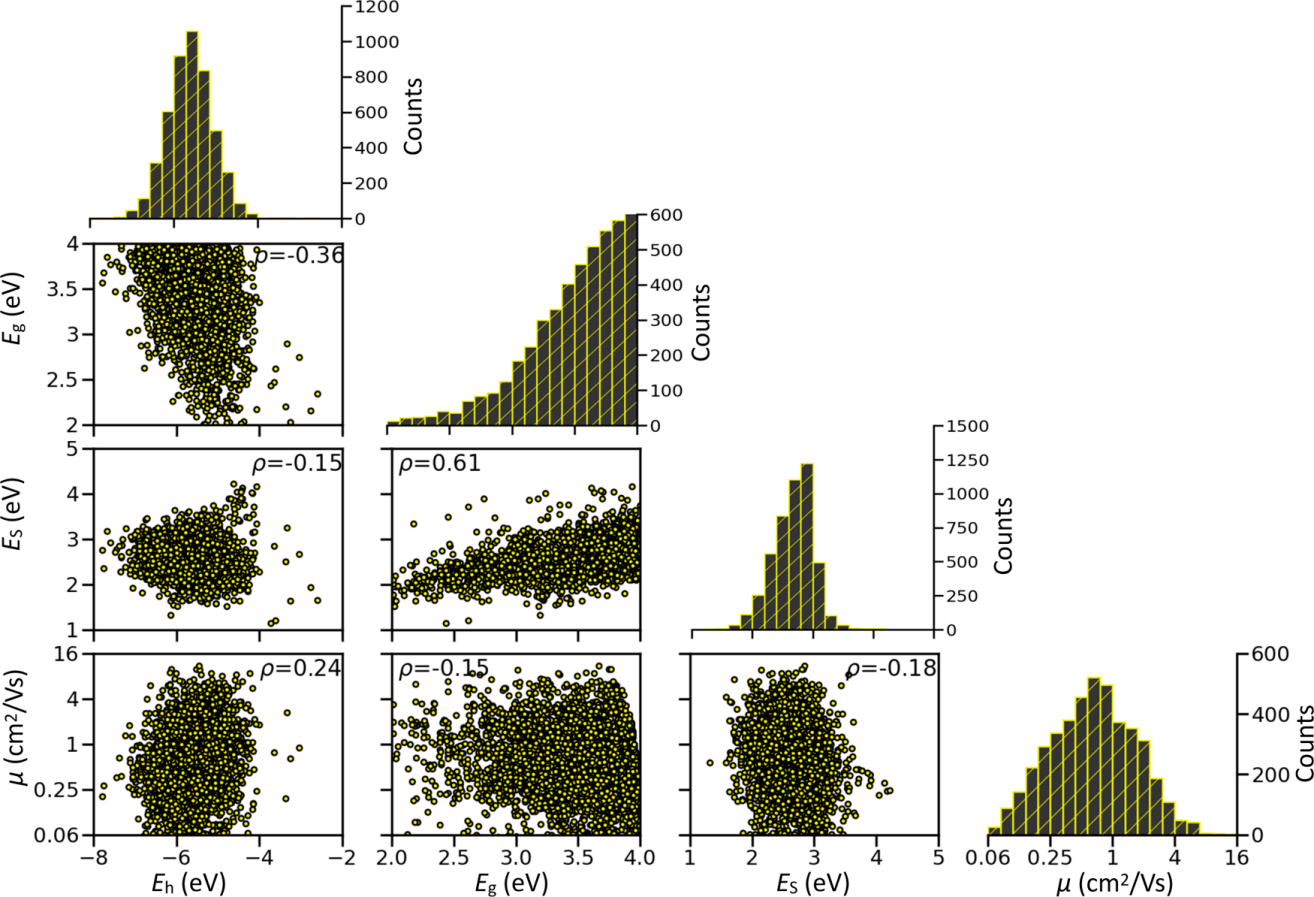
Distributions of TCM parameters alongside scatter plots representing the relations between them. Spearman’s rank correlation ρ values are given in the insets.

The key parameters to consider for the screening are, therefore, *E*_S_ for the transparency, μ for the charge-transport properties, and *E*_h_ for the ability to be p-dopable. It is also useful to analyze how these quantities are related to the computed *E*_g_. A useful first step to rationalize the results is considering the distribution of these quantities and their correlations, which are illustrated in Figure 2 (a two-dimensional (2D) heatmap version of this figure as well as the 95% confidence interval for all of the correlation values can be found in the SI). From the diagram, it is possible to see, for example, that the HOMO energy levels follow a normal distribution and there is a considerable fraction of materials in the database that would be easily dopable as they possess the high-lying HOMO *E*_h_ energy level (see below for the definition of a reasonable cutoff). According to the distribution of computed mobility, more than 31% of the materials considered have μ exceeding 1 cm^2^/Vs and can be therefore of interest to TCMs. It should be noted that this fraction is high because the considered 4801 solids have been obtained from the original data set by excluding those with expected low mobility. Much more stringent appears to be the criterion of transparency, i.e., *E*_S_ ≥ 3.26 eV, as the number of molecules with large *E*_S_ values decreases drastically, highlighting the scarcity of transparent materials in comparison with dopable ones.

There is a noticeable correlation, observed in Figure 2, between the HOMO–LUMO gap *E*_g_ and the energy of the lowest allowed excited state *E*_S_, but the correlation is too weak to allow prediction of transparency from the *E*_g_. For example, molecules with *E*_g_ in the [3.0, 3.2] eV range display a very broad range of *E*_S_ between 1.3 and 4.0 (with average and standard deviation of 2.6 and 0.27 eV, respectively). Similarly, broad distribution is found if the energy of the S_1_ excitation state instead of *E*_S_ is considered (see the Supporting Information). The second-largest correlation, comparatively much smaller, is between *E*_g_ and *E*_h_, and it is obviously expected from the definition of *E*_g_ as the difference between LUMO energy and *E*_h_. Figure 2 also shows that there is a very limited correlation between mobility and transparency with a Spearman correlation coefficient ρ being only −0.18 and the 95% confidence interval [−0.20, −0.15]. The practical consequence is that one should search for materials that satisfy both conditions at the same time as the degree of correlation is too low. Similarly, the correlations between the rest of the parameters are also not remarkable, and one can think of them as independent variables in materials optimization.

To identify promising structures that can be used in transparent electronics, one needs to first set realistic thresholds for physical parameters. It is important to note that these thresholds are not absolute because of computational inaccuracies and the fact that one may give different weights to the different criteria, but they are useful to discuss the number and type of molecules that one is expected to find, which is one of the goals of this paper. To establish a reasonable threshold for *E*_h_ and *E*_g_ we collected in Table 1, a sample of materials displaying high experimental mobility (μ ≥ 0.5 cm^2^/Vs) with the corresponding values of *E*_h_ and *E*_g_ computed at the same level used for the database of molecular crystals. According to the table, the computed HOMO energy level of the considered structures is primarily in the range of [−5.6, −4.6] eV with a HOMO–LUMO gap of [1.4, 3.9] eV. Therefore, to find dopable structures, we focus on those possessing HOMO level larger than −5.6 eV. We do not set any limitation on the *E*_g_ values as the largest observed in Table 1 (3.9 eV) is already very close to the one in our considered database (4 eV). A particularly important observation of the materials listed in Table 1 is that, in accordance with the findings of Figure 2 that highlights the negligibility of the correlation between mobility and transparency, none of these materials is transparent in the visible range, highlighting the difficulties in finding transparent materials from a common set of high-mobility molecular semiconductors.

The natural criterion for the lowest allowed transition *E*_S_ is to consider molecules for which this parameter is larger than 3.26 eV, the conventional visible/ultraviolet boundary. However, computed excitation energies are subject to computational error, and the absorption can be shifted from violet to ultraviolet by introducing small chemical modifications.^110−112^ Considering these points and also the fact that there is a sharp decrease in the fraction of molecules with *E*_S_ ≥ 3 eV, as shown in Figure 2, it is useful to consider in detail all molecules with *E*_S_ ≥ 3 eV as it is easier to identify patterns from larger data sets and, in this way, no interesting molecule can be missed. Using these thresholds, one can see (Table 2) that about half of the structures are dopable (49%). The reason is that the criterion for selecting a molecule as a semiconductor (small HOMO–LUMO gap) is akin to that of the dopability and, as explained above, materials with small *E*_g_ are expected to possess a higher-lying HOMO energy level as well. Transparency is a much stringent criterion (13% satisfy the criterion *E*_S_ ≥ 3 eV and 2.3% the criterion *E*_S_ ≥ 3.26 eV), and it is therefore much more difficult to find transparent materials among molecular semiconductors. The combination of dopability and transparency follows the statistics of uncorrelated properties. It is instructive to verify the original hypothesis of this work on the transparency of molecular semiconductors. According to our analysis, of the 650 transparent molecules, 25% are transparent due to the existence of forbidden transitions to the lower excited states such that 17.8% had one and 7.2% had two or more forbidden states below the 3 eV threshold. In the remaining 75%, the first singlet excited state is already above the threshold, more common in molecules with a small *E*_g_ – *E*_S_.

**Table 2:** Number/Percentage of Structures Satisfying Required Criteria as Potential TCMs

criteria	thresholds	number of structures	percentage (%)
entire database		4801	100
dopable	(A) *E*_h_ ≥ −5.6 eV	2354	49
transparent	(B) *E*_S_ ≥ 3 eV	650	13
dopable and transparent	(A) and (B)	299	6.2
dopable, transparent, and high mobility	(A) and (B) and μ ≥ 1 cm^2^/Vs	81	1.7

Among the identified 299 potential TCM materials, 81 structures have computed mobility larger than 1 cm^2^/Vs, and their full list can be found in Table S2 in the SI. An important observation is that the candidates are quite diverse and cannot fall within a limited number of chemical classes, which is a very positive outcome for a virtual screening as it indicates that there are more leads for a potential discovery. This is a consequence of having used a set for testing that was not built to have certain properties and that can be considered “unbiased” in this sense. Furthermore, we note that we have neglected, for simplicity, the effect of crystal packing on the absorption edge as spectral shifts in absorption edges are typically on the order of ∼0.1 eV (0.11 eV is the median value found among considering only bright excitons^62^). Our analysis reveals that the oscillator strength of identified promising molecules, with a median being 0.12, is considerably smaller than those of molecules considered in ref (62), which result in small excitonic couplings. As reported in the SI, the median of the largest excitonic coupling in this database, considering an average dielectric constant 3,^114^ is 0.0117 eV with a maximum being 0.0928 eV, implying that these structures have very narrow excitonic bandwidths and retain most of molecular characteristics. Therefore, the impact of crystal packing on the absorption properties can be simply neglected. Ultimately, all of the identified 81 molecules continue to satisfy the criteria for TCM after optimization of the geometry, as shown in the SI. Furthermore, in the SI, we show that there is a moderate correlation between the charge-transfer integrals and excitonic couplings.

The condition of having high mobility removes from consideration many zwitterionic molecules (shown in Table S3), which meet the criteria of dopability because of the anionic fragment and the transparency because the lowest charge-transfer transitions are typically forbidden.^115^ Zwitterions, however, are not leading to high mobility and are not promising candidates for TCMs because the excess charge is too localized and, consequently, the local electron–phonon coupling is large.^116^

Among the 81 structures meeting all of the desirable criteria, only a minority of them, i.e., 12 molecules, have a forbidden state in the gap, which was different from the expectation (Figure 1), and has implication on the design of TCMs. The possible origin of forbidden (or quasi forbidden) transitions includes the charge-transfer nature of transition^117^ or symmetry.^118^ To analyze the origin of such low-intensity transitions, we used the Δ*r* index, as introduced in ref (119) and implemented in the multi-wave-function package,^120^ which measures the charge-transfer length associated with a given transition. This index, in other words, helps to quantify the charge-transfer nature of a transition. According to this analysis, in nine molecules from this set, the charge-transfer nature of the transition, with Δ*r* index being larger than 1.8 Å, is the main reason for their very low absorption intensity in the visible. For the other three molecules, the transition is not strictly forbidden by symmetry, but very low oscillator strengths can also be observed in non-charge-transfer transition for a data set of this size. It is notable that although the presence of forbidden transitions did not turn out to be the main player in the transparency of the identified high-performance material, this relation is worth mentioning because molecules can be designed to have charge-transfer character^113^ and therefore can make the combination of small band gap and transparency feasible.

**Figure 3:**
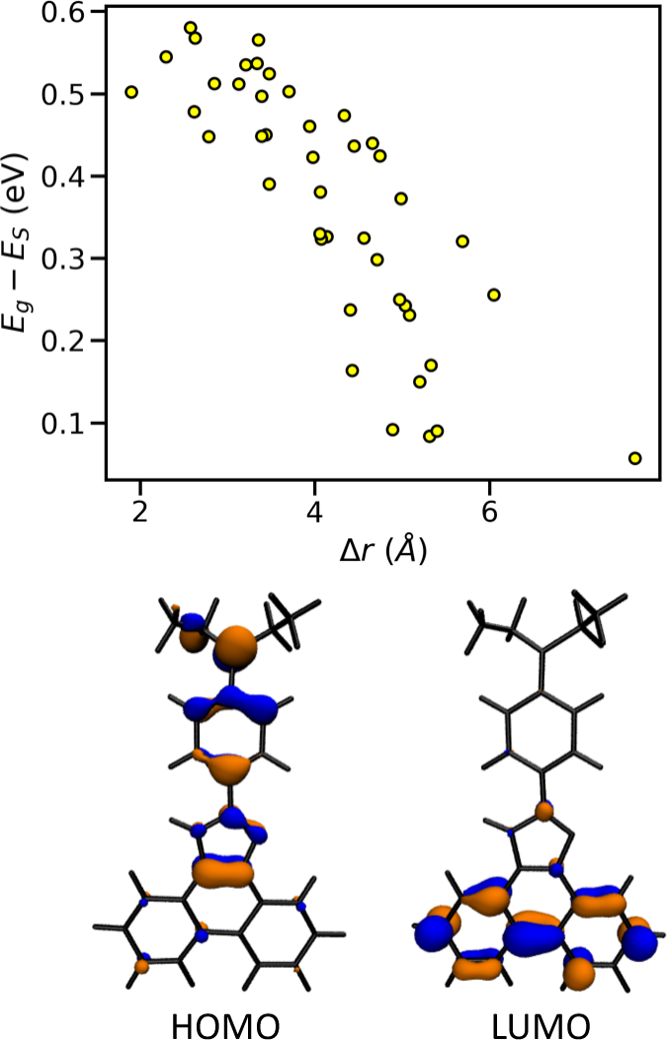
(Top) Scatter plot showing the variation of the energy difference between *E*_g_ and *E*_s_ against the charge-transfer length during electron excitation Δ*r*. (Bottom) DFT-computed density of frontier orbitals involved in excited-state transition of charge-transfer nature.

Considering the 69 molecules with the first excited state above the visible gap, we observed that the median energy difference *E*_g_ and *E*_S_ in this set of molecules is 0.44 eV, considerably smaller than the median value of the initial database 0.89 eV. Our analysis shows that in 49 of these molecules, where *E*_g_ – *E*_S_ is smaller than 0.55 eV, the excited-state transitions are of a charge-transfer nature (82% of them are dominated by HOMO to LUMO transition). As shown in Figure 3, the energy difference of *E*_g_ – *E*_S_ decreases with increasing Δ*r*, i.e., with the increase of the charge-transfer nature of the transition and the corresponding reduction of the HOMO–LUMO exchange energy. In essence, a charge-transfer nature of the transition brings the excited singlet energy closer to the *E*_g_ value. The figure exemplifies the spatially separated frontier orbitals of a molecule with Δ*r* = 4.9 Å. The remaining 20 molecules for which one cannot label the lowest excited state as a charge-transfer state are less remarkable: they have a relatively large HOMO–LUMO gap to start with (i.e., *E*_g_ ≥ 3.8 eV) and remain transparent because their energy difference *E*_g_ – *E*_S_ is never larger than a moderate value of 0.75 eV. These findings are further supported by the results of a nonparametric Mann–Whitney U test,^121^ with a *p* value being smaller than the reference value 0.05, which highlights the difference between the distribution of *E*_g_ – *E*_S_ in the database of transparent high-mobility materials and in the full database.

**Figure 4:**
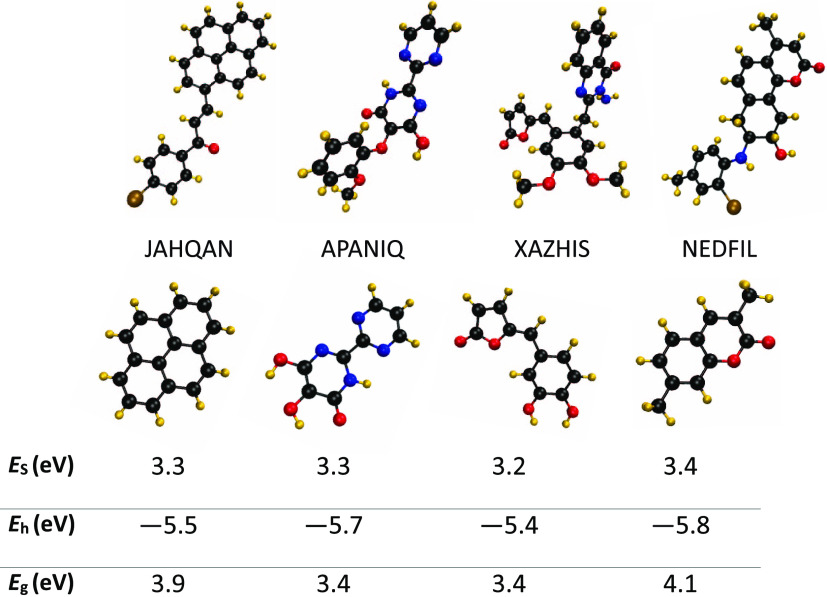
Molecular structure of some of the promising TCMs (top row) and proposed lead compounds derived from them (bottom row). The values of physical parameters of smaller fragments given in the bottom row are summarized in the given table. Molecules are labeled with their CSD identifiers.

It is useful for practical applications to find out if smaller fragments of the identified promising TCMs retain the same electronic properties on their own, i.e., they can be used as lead compounds for future materials exploration. To this aim, in Figure 4, we provide representative examples where the small fragments are built from their parent molecules labeled with their CSD identifier, and their optimized geometry has been used for electronic structure calculations. The first molecule, identified as JAHQAN in CSD, is a derivative of pyrene, also shown underneath in the second row, which is indeed transparent and well studied in the literature.^122^ There are almost 45 compounds containing pyrene in our initial database of semiconductors extracted from CSD. The majority of them, however, did not make it into the database of materials with desirable charge-transport properties due to their weak electronic couplings. As there are ways to engineer crystals to boost their charge-transport characteristics,^79,123−126^ the pyrene derivatives can be considered as potentially promising structures for transparent electrodes. The remaining three molecules, identified as APANIQ, XAZHIS, and NEDFIL, are also promising as their transition is of charge-transfer nature, leading to small exchange energies. APANIQ and NEDFIL contain, respectively, pyrimidine and coumarin. There are rare studies in the literature that have also noted the potential transparency of pyrimidine as well as coumarin derivatives^127,128^ that is associated with the fact that their local π–π excitation dominates their main type of electronic transition. There are not any discussions on the transparency of XAZHIS, which highlights the fact that high-throughput screening studies are able to find molecules that are not initially synthesized for the applications intended by the screening. Furthermore, we found that there are not huge differences between the best p-type organic and inorganic TCMs, such that the largest mobility which is reported for a family of the best inorganic p-type TCMs is ∼21 cm^2^/Vs, which is associated with SnO.^129^ The largest mobility in the database of 81 potential organic TCMs identified in this work is ∼9 cm^2^/Vs, which belongs to JAHQAN. The mobility reported for other p-type inorganic TCMs is mostly below 10 cm^2^/Vs,^130^ which is comparable to those of our database. When comparing organic and inorganic TCMs, one can notice that different chemical classes can be preferred for different applications in relation with the processing methods and the compatibility with other materials in the devices. Furthermore, considering the practical strategies that have been proposed for increasing mobility in molecular semiconductors,^79,125^ it is expected that the performance of molecular semiconductors as potential TCMs can be even further improved.

Interestingly, we found that the smaller fragments of the best molecule identified in this work, shown underneath in the second row of Figure 4, have similar electronic properties, leading them to be transparent with their physical parameters summarized in the lowest panel of the same figure. These examples highlight the fact that the identified promising TCMs are clearly not the only molecules with desirable characteristics: they can be used to initiate new lines of investigations where their small fragments or slightly modified chemical structures can be evaluated for suitability for use in transparent electrodes.

These findings alongside the distribution of properties given in Figure 2 suggest an optimal strategy to identify novel TCMs through virtual screening. One should focus on the most stringent condition, transparency, which requires excited-state calculation but remains a property of the molecule rather than the material (assessing for dopability comes at no additional computational cost). As the data utilized in this work was extracted from the world’s largest repository of small organic molecules with experimentally known crystal structures (CSD),^76^ future studies presumably would focus on molecules or hypothetical molecules with unknown crystalline structure. This being the case, to limit the search space to those with potentially high mobility, molecules can be first screened for low-oxidation reorganization energy, the molecular property with the highest correlation with solid-state charge mobility,^31^ and then the crystal structure prediction methods^131^ can be applied to the promising ones.

## 4 Conclusions

Many attempts are being made to identify and develop new p-type transparent conducting materials for a variety of applications and devices. In this demanding process, computationally driven materials discovery and design play a vital role. We screened the Cambridge Structural Database to investigate the likelihood of transparent conducting materials technology based on p-type-doped molecular crystals. The main insight of this work is that a technology based on organic TCMs is feasible and a good number of candidate materials—already characterized in the solid state—can be identified from virtual screening. As such, we found that the number of structures that simultaneously meet the criteria of transparency, dopability, and high mobility is limited, and yet the discovered 81 potential high-performance materials constitute a firm ground for experimental investigations. Our results showed that molecular semiconductors with HOMO–LUMO gap larger than the range of visible spectrum become transparent if their HOMO–LUMO exchange energy is small enough to preserve the energy of the lowest allowed transition above the visible spectrum. Those with a small HOMO–LUMO gap, however, can also become transparent if their lowest energy excited state(s) are optically forbidden. The former scenario was found to be the prevalent case in this material class; however, the latter one is also of practical importance. There is a known relation between low oscillator strength and charge-transfer character; therefore, molecules can be designed to have charge-transfer character making the combination of small band gap and transparency feasible. The charge-transfer nature of electronic excitations was also found to play an important role in leading excitation energy above the visible gap. Such findings open a new perspective to TCM design and discovery motivating further detailed research on how to develop high-mobility molecules with charge-transfer excited-state transitions. More promising candidates can be proposed by identifying the smallest molecular fragment of the promising TCM molecules that retain similar key electronic properties: such fragment can be used as a lead compound for follow-up investigations. Finally, considering the drawn insights from the identified promising materials as well as the distribution of physical parameters, we proposed a strategy illustrating what would be a practical method to discover novel compounds potentially with an unknown crystalline structure. This study illustrates how high-throughput virtual screening can be particularly beneficial for the search of materials with an unusual combination of properties with high technological significance to assess whether such materials exist and how rare they are. Such studies produce both lead candidates for future experimental investigation and strategies for new virtual explorations.

## Supporting Information Available

Comparison of frontier orbitals- and excited-state energies computed on experimental and optimized geometries; justification of threshold of oscillator strength; the energy difference between the HOMO–LUMO gap and the first singlet energy; the molecular diagram of potential TCMs; the molecular diagram of transparent dopable Zwitterions; and data availability (PDF)
