# Supplementary material for: Feasibility of p-Doped Molecular Crystals as Transparent Conductive Electrodes via Virtual Screening
Source: Chem Mater. 2022 Apr 25;34(9):4050–61. doi: 10.1021/acs.chemmater.2c00281 (PMC9097283; doi:10.1021/acs.chemmater.2c00281)
Supplement: Supplementary file 1 — cm2c00281_si_001.pdf [file cm2c00281_si_001.pdf]

SUPPORTING INFORMATION FOR:

Feasibility of p-doped molecular crystals as transparent conductive electrodes via virtual screening

Tahereh Nematiaram, Alessandro Troisi

Dept. of Chemistry and Materials Innovation Factory, University of Liverpool, Liverpool L69 7ZD, U.K.

tahereh.nematiaram@liverpool.ac.uk; a.troisi@liverpool.ac.uk

Contents:

A. Comparison of frontier orbitals- and excited state energies computed on experimental and optimized geometries

B. Justification of threshold of oscillator strength

C. Excited states of the cation

D. The energy difference between the bandgap and the first singlet energy

E. Calculations of excitonic couplings

F. Relation between excitonic couplings and charge transfer integrals

G. A 2D-heatmap showing the relation between physical parameters relevant to TCMs

H. The molecular diagram of potential TCMs

I. The molecular diagram of transparent dopable Zwitterions

J. Data availability

References

A. Comparison of frontier orbitals energies computed on experimental and optimized geometries

The calculations related to dopability and transparency in this work have been performed on experimental geometry. Therefore, it is important to figure out to what extent the key characteristics can be retained if the geometry is optimized. To this aim in Figure S1, a comparison between the frontier orbitals energies of the identified 81 promising molecules utilizing experimental (Xray) and optimized geometries has been provided. The geometry optimization is done using the procedure developed for the large scale screening conducted in Ref.[1]. As can be seen, the range of orbitals energies on both geometries is similar and, in particular, the cut-off for dopability, requiring the

HOMO energy level being larger than −5.6 eV, is fully fulfilled by both optimized and experimental geometries.

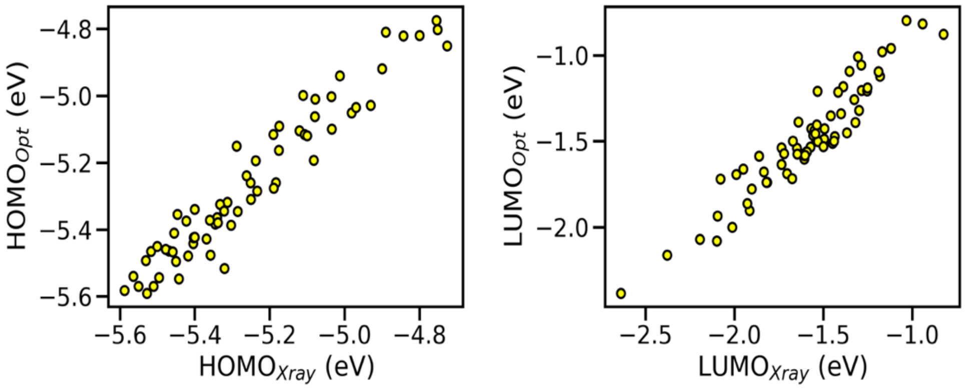

Figure S1. A scatter plot showing the relation between frontier orbital energies computed on experimental (Xray) and optimized (Opt) geometries.

The same kind of analysis is also performed for the excited state energies as shown in Figure S2 which again manifests a similar range of values for the first singlet energy computed on experimental and optimized geometries. Of particular importance is the fact that, all the identified 81 molecules continue to satisfy the criteria for transparency after optimization of the geometry. This highlights the fact the calculations on experimental geometries provide reliable results.

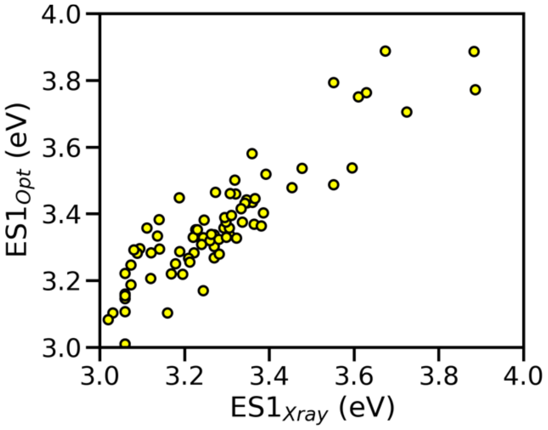

Figure S2. A scatter plot showing the relation between the singlet energy computed on experimental (Xray) and optimized (Opt) geometries. The energies are calibrated using the formula given in the main manuscript.

B. Justification of threshold of oscillator strength

A thin layer is considered transparent if its transmittance is above ~90%. The absorption coefficients of many inorganic semiconductors are in the range of 10^4—10^5 cm^{-1}, meaning that if a film is 200 nm thick (which is considered relatively thin), the transparency will be between 82% ($= exp^{-0.2}$) and 14% ($= exp^{-2}$). Accordingly, films with small thickness will be transparent and thicker ones will be opaque. The same range of absorption coefficients is seen in organic semiconductors, e.g., absorption coefficient in rubrene is 0.5×10^5 cm^{-1}, as reported in Ref.[2], and it is slightly larger in pentacene,[3] and

similar values are reported for other semiconductors.[4,5] Therefore, a 1000 nm rubrene or pentacene film have transparency 0.6% ($= exp^{-5}$). The oscillator strength of Rubrene is computed to be 0.21 by us in close agreement with the value reported in ref.[1] but only relatively consistent with those reported in ref.[2] obtaining 0.14, 0.28, 0.37 employing different level of theories. The absorption coefficient of Rubrene is shown to be 0.5×10^5 cm^{-1}.[3] As the optical absorption have similar width (within a factor of 2-3) we can assume that the absorption coefficient is approximately proportional to the oscillator strength. Therefore, considering f=0.21 as our reference, a film of 1000 nm thickness and largest oscillator strength lower than 0.0005 will have transparency exceeding 98.8%. This criterion could be relaxed to f lower than 0.002 to shift down the threshold of transparency to 95% increasing the number of molecules to consider from 650 to 669 implying the results in the main manuscript are not much affected by the choice of this threshold. This explains why setting the oscillator strength to 0.0005, in the screening, albeit arbitrary is reasonable. We have also neglected the carriers and dopants absorption assuming that they have the same typical absorption coefficient (0.5×10^5 cm^{-1}), reduced by a plausible doping level of (2%). A 1000 nm (500nm) thick film will then have ~90% (95%) transparency because of the absorption of the dilute carrier. This is the reason why carrier absorption is neglected in this work and in all previous works based on inorganic semiconductors (having similar absorption coefficient).

C. Excited states of the cation

In the main manuscript we estimated that, if oxidized molecules absorb in the visible, they are not expected to influence the transparency of the film at typical concentration and film thicknesses. To be more quantitative we computed explicitly the excited states and corresponding oscillator strength for the molecular cation using the same methodology used for the neutral species (and optimized geometry of the cation). We have included 12 doublet excited states to ensure the last one(s) included is always outside the visible spectrum.

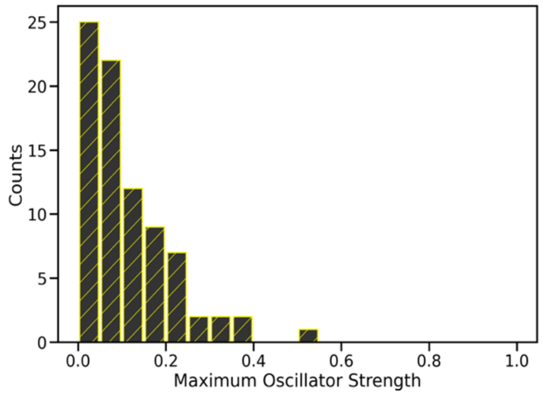

Figure S3. The distribution of the maximum oscillator strength of cations in the visible spectrum.

In Figure S3, the distribution of the largest oscillator strength f of cations in the visible range, for the database of 81 high performance materials, is shown. As can be seen, the median value of f is 0.071, 1.1 larger than in pentacene, whose molecular volume is slightly smaller than the median of the dataset (it has 22 heavy atoms with the median value of the TCMs dataset being 27). The transparency is given by $\exp(-\alpha T)$ with α and T being, respectively, the extinction coefficient and film thickness. The median extinction coefficient can be estimated as 1.1 times the extinction coefficient of pentacene at it maximum ~0.7×10^5 cm^{-1}.[3] Therefore, for a typical doping concentration of 2%, typical thickness of 500 nm, the transparency can be estimated as 93.2% ($exp^{-(0.02\times0.7\times5)}$). Approximately 53 out of 81 proposed TCMs have transparency greater than 90% with this thickness and doping concentration; only 14 out of 81 will have transparency below 90% at 1% doping concentration and the same thickness; and all are transparent at a doping concentration of 1% and film thickness of 200 nm. The cation oscillator strength is reported in the repository.

D. The energy difference between the bandgap and the first singlet energy

Our results indicated that the bandgap cannot be reliably used to predict the transparency in molecular semiconductors. Figure S4 represent the energy difference between the bandgap E_g and the first singlet energy E_{S1} plotted versus different values of E_g. As can be seen this difference in energies can be really broad, and in order to predict the transparency in this materials class, one needs to necessarily perform the exited state calculations.

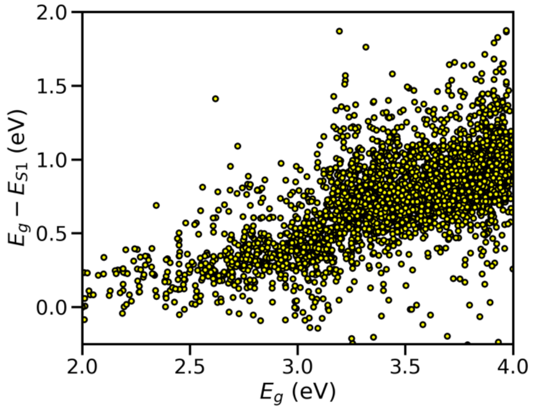

Figure S4. The variation of energy difference between E_g and E_{s1} against electronic bandgap.

E. Calculations of excitonic couplings

For the calculations of excitonic couplings the methodology of Ref.[6] is utilized and the resulting values are reported in the attached dataset (see section G). As can be seen, the median of the largest excitonic coupling in this database, considering an average dielectric constant 3,[7] is 0.0117 eV with a maximum being 0.0928 eV implying that the molecules identified as potential TCMs have very narrow

excitonic bandwidths and retain most of molecular characteristics. Therefore, the impact of crystal packing on the absorption properties is neglected.

F. Relation between excitonic couplings and charge transfer integrals

It is also informative to figure out if there a correlation between charge transfer integrals and excitonic couplings. In our previous works, the charge transfer integrals have been computed for all the molecular semiconductors in the Cambridge Structural Database (i.e., ~40,000 structures). The excitonic couplings are computed only for those composed of molecules whose lowest excited states are strongly coupled and generate wide excitonic bands (i.e., ~2200 structures). To make a reasonable comparison between the two sets, in Figure S5, we show that the distribution of the largest charge transfer integral (J_1) for (a) the whole dataset, (b) for structures with computed excitonic couplings, and (c) ultimately for those exhibiting wide excitonic bands (i.e., structures with excitonic bands larger than 0.5 eV considering an average dielectric constant 3). As can be seen, the median of J_1 in these instances increases from 0.038 eV to 0.048 eV and ultimately to 0.077 eV. Accordingly, one can conclude that the charge transfer integral is expected to be slightly larger in materials whose lowest excited states are strongly coupled and particularly in those who generate very wide excitonic bands. However, the correlation between excitonic and charge transfer coupling is very modest as one can appreciate from the broad distribution shown in panel (c) below.

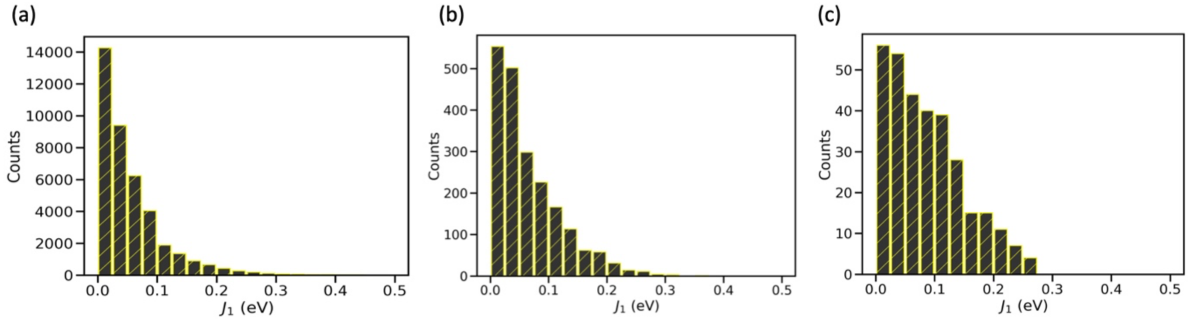

Figure S5. The distribution of the largest charge transfer integral for (a) the whole dataset, (b) for structures with computed excitonic couplings, and (c) for those exhibiting wide excitonic bands.

G. A 2D-heatmap showing the relation between physical parameters relevant to TCMs

In this section, a 2D heatmap version of the Figure 2 of the main manuscript is shown. Furthermore, we have summarized the spearman rank correlation values between physical parameters relevant to TCMs, i.e., the HOMO energy (E_h), HOMO-LUMO gap (E_g), the energy of lowest allowed transition (E_S), and the charge mobility (μ), as well as their 95% confidence interval.

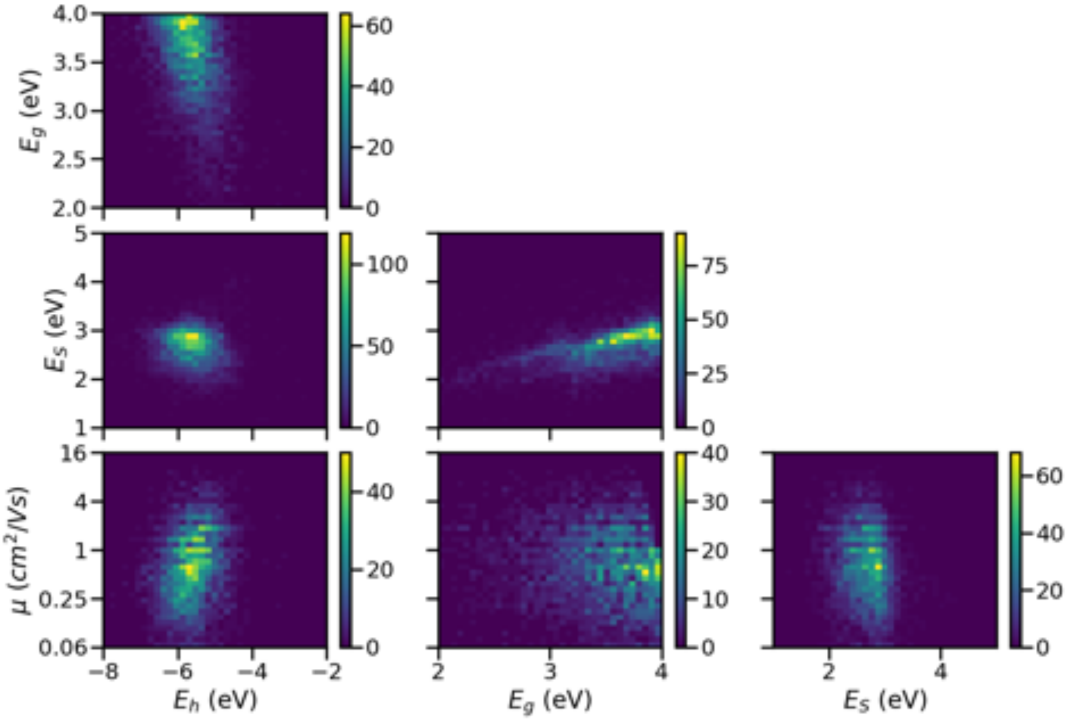

Figure S6. A 2D-heatmap showing the relation between physical parameters relevant to TCMs, i.e., the HOMO energy (E_h), HOMO-LUMO gap (E_g), the energy of lowest allowed transition (E_S), and the charge mobility (μ).

Table S1. The 95% confidence interval for all the spearman ρ values highlighting the correlation between the parameters relevant to TCMs, i.e., the HOMO energy (E_h), HOMO-LUMO gap (E_g), the energy of lowest allowed transition (E_S), and the charge mobility (μ).

Parameters	Correlation	95% confidence interval
E_g and E_h	−0.36	[−0.38, −0.33]
E_S and E_h	−0.15	[−0.17, −0.12]
E_S and E_g	+0.61	[+0.59, +0.63]
μ and E_h	+0.24	[+0.21, +0.27]
μ and E_g	−0.15	[−0.18, −0.12]
μ and E_S	−0.18	[−0.21, −0.15]

H.

Table S2. The molecular diagram of potential TCMs.

1	2	3	4
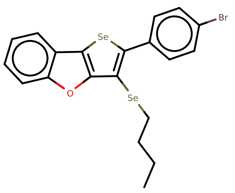	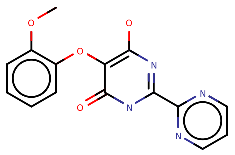	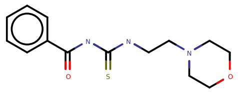	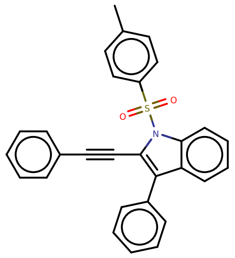
ABENED	APANIQ	AVAPEU	BEBGOG
**5**	**6**	**7**	**8**
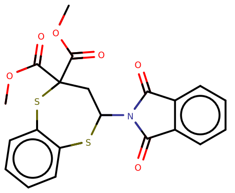	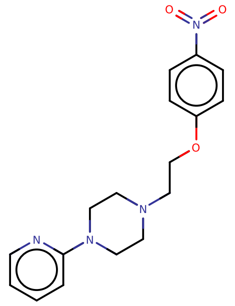	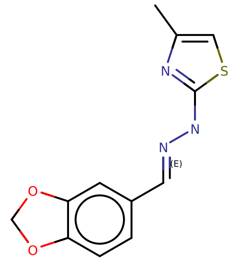	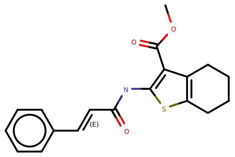
BUWHUY	CAYTOO	EFATUA	EVIGUL
**9**	**10**	**11**	**12**
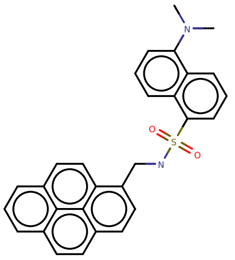	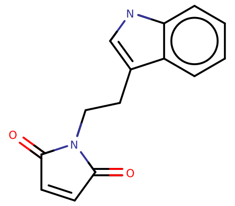	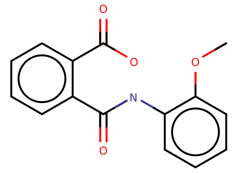	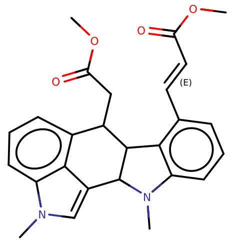
EZEVIP	FEGSIW	FICLAG	FICPOZ
**13**	**14**	**15**	**16**
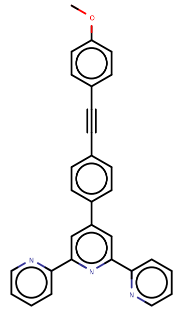	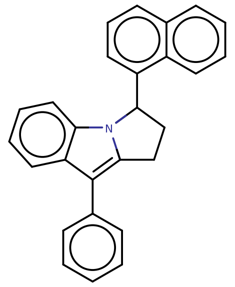	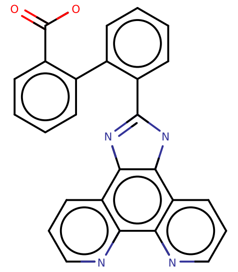	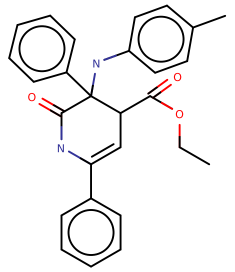
FONKOK	GEGKOU	GEQBIP	GIHZOM

17	18	19	20
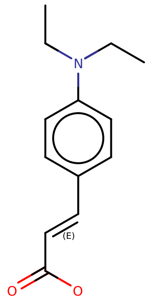	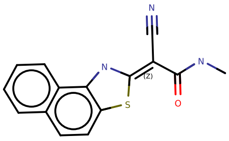	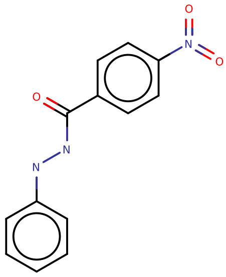	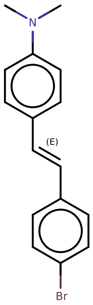
GILNEW	GIYZEU	HIFRAQ	HOZDAC
**21**	**22**	**23**	**24**
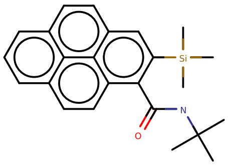	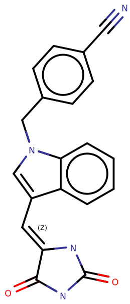	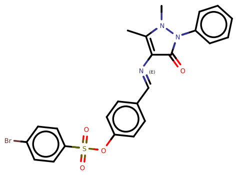	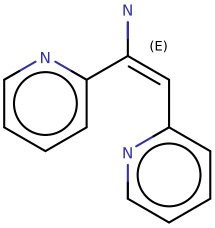
HUTLOZ	IFUXAJ	IGAQEN	IGIFOT
**25**	**26**	**27**	**28**
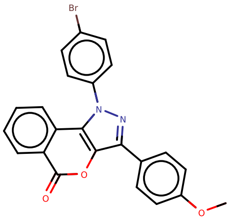	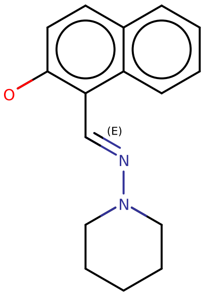	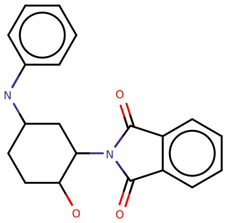	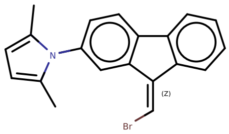
IKIGIU	IMUREO	IRUXEZ	IVEXOY
**29**	**30**	**31**	**32**
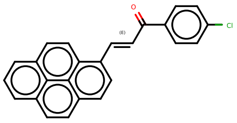	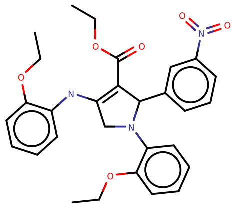	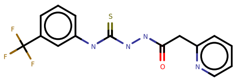	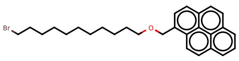
JAHQAN	KARKEV	KAXYUG	KEKWON

33	34	35	36
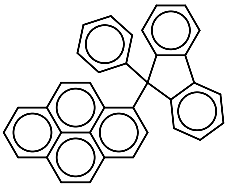	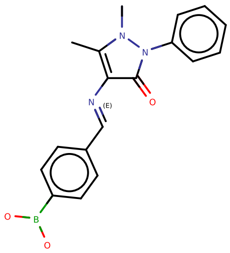	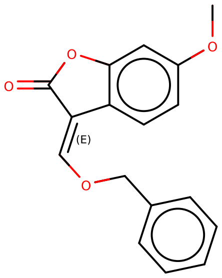	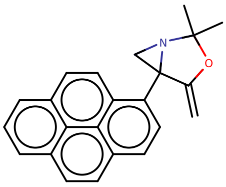
KENXAD	KERQOP	KIYCIE	KUGFEY
**37**	**38**	**39**	**40**
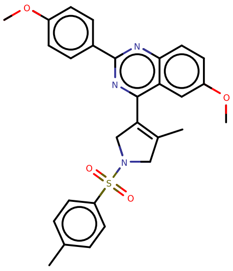	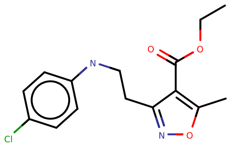	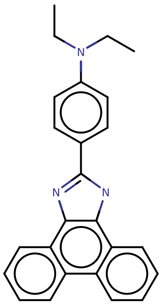	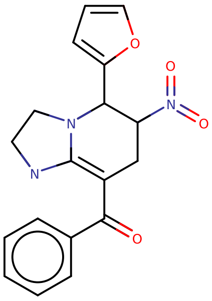
LIBTIC	LUCYOZ	MORMAH01	MUCCIX
**41**	**42**	**43**	**44**
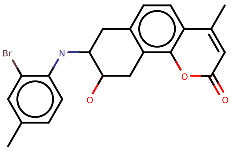	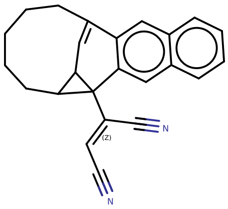	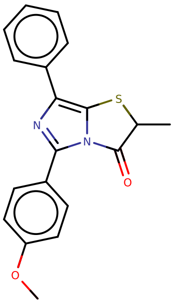	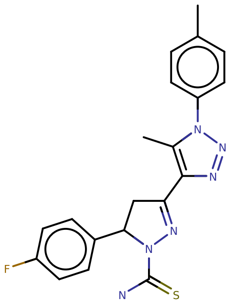
NEDFIL	PAGRIY	PASKUQ	PAXREN
**45**	**46**	**47**	**48**
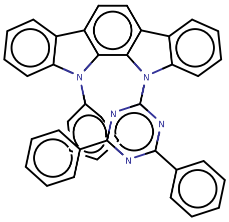	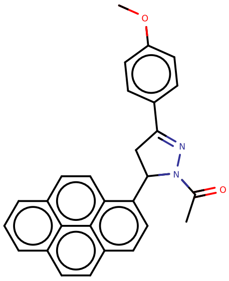	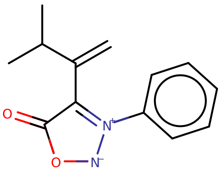	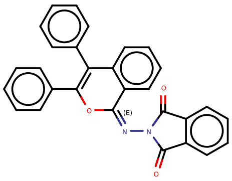
PERXUI	PESHEC	PMBSYD	PUVNOL

49	50	51	52
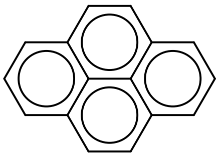	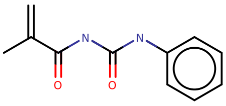	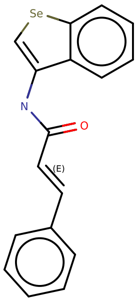	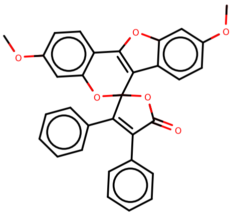
PYRENE03	QAZXIY	QIRFEE	QIWMUG
**53**	**54**	**55**	**56**
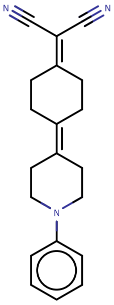	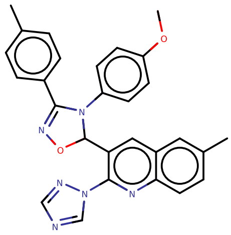	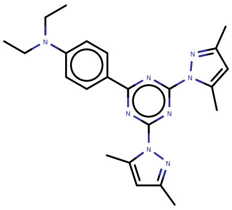	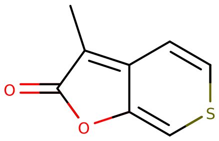
QUGKUY	QUMSIC	RUQSEC	SOSBAF
**57**	**58**	**59**	**60**
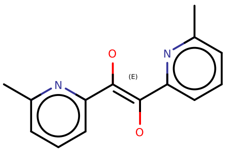	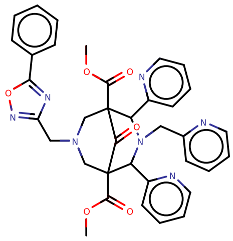	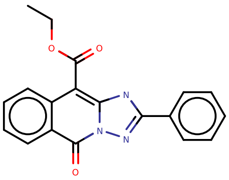	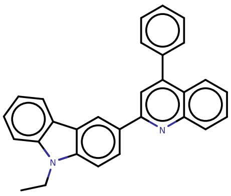
TEBMIX	TIQQIV	TOFTOZ	TOPGIP
**61**	**62**	**63**	**64**
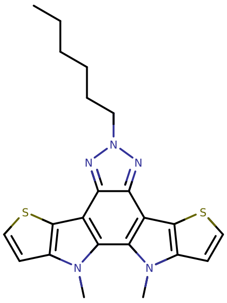	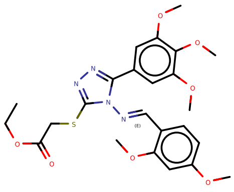	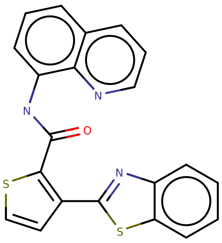	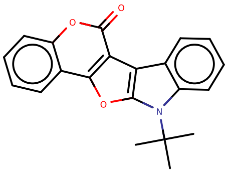
TOSMEV	UQAPIM	UVUGOJ	UXUKOO

65	66	67	68
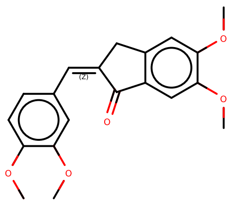	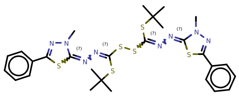	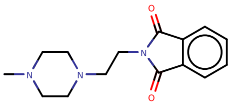	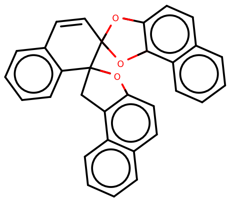
VABMAO	VAHYAE	VITTID	WATBAU
**69**	**70**	**71**	**72**
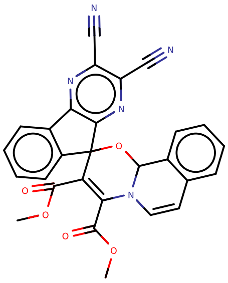	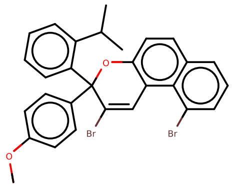	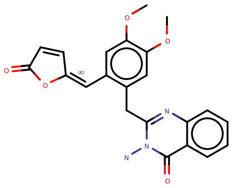	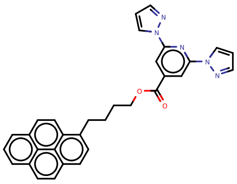
WEMVIW	WOYSUA	XAZHIS	XAZKER
**73**	**74**	**75**	**76**
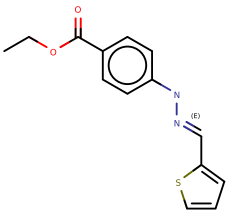	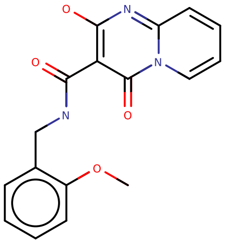	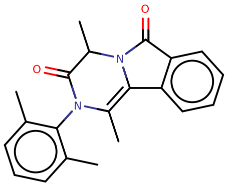	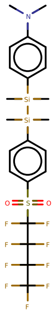
XIGHUS	XOQHIV	YADRUT	YATNEM
**77**	**78**	**79**	**80**
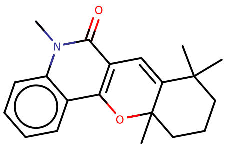	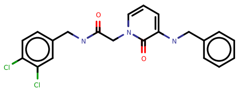	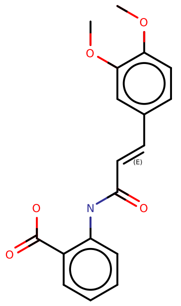	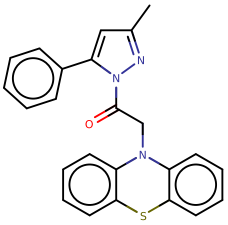
YIBBET	YIKNAJ	YIPFUY01	ZILRAP

81			
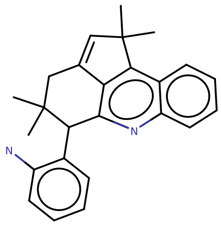			
ZORFUH			

I.

Table 3. The molecular diagram of transparent dopable Zwitterions in the database.

1	2	3	4
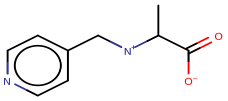	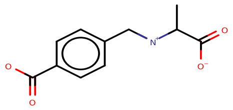	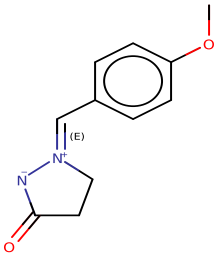	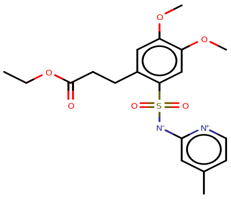
BEPMOA	BETJER	BOSXOW	BULFOD
**5**	**6**	**7**	**8**
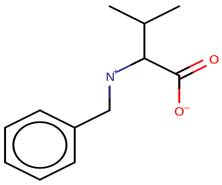	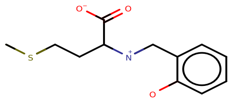	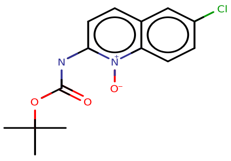	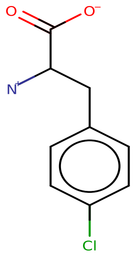
COLWAD	EROGEY	EWEHUL	EWUZED
**9**	**10**	**11**	**12**
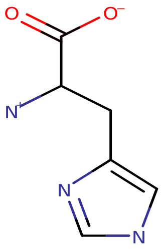	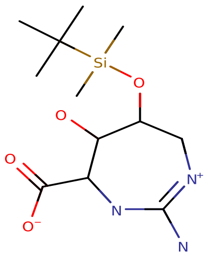	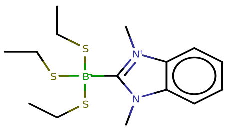	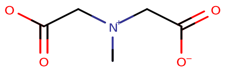
FAJMIP	FAKWEV	FEFNOW	FENTOH
**13**	**14**	**15**	**16**
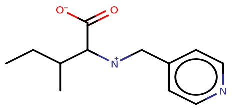	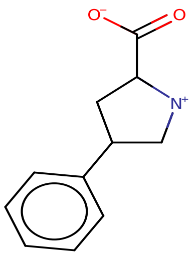	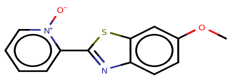	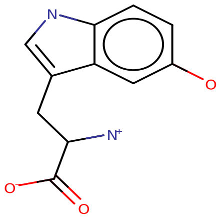
GASRAW	GAYCEO	GIPBAK	HTRYPT10

17	18	19	20
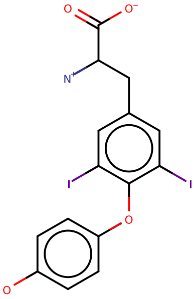	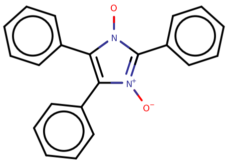	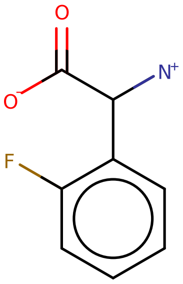	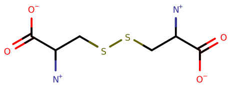
ITUBEG	JADNAE	LALXAX	LCYSTI10

21	22	23	24
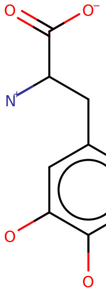	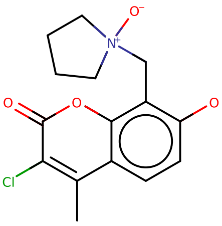	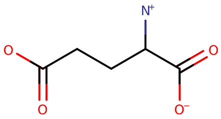	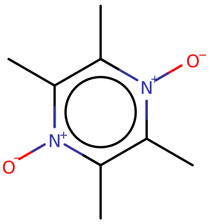
LDOPAS11	LEJTUR	LGLUAC11	LOXFUB

25	26	27	28
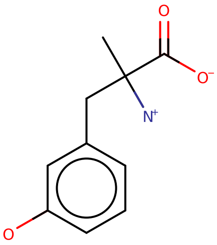	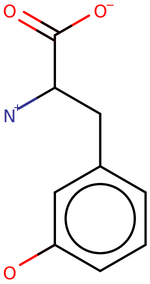	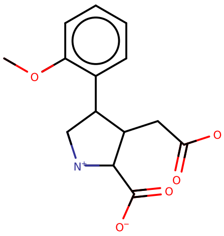	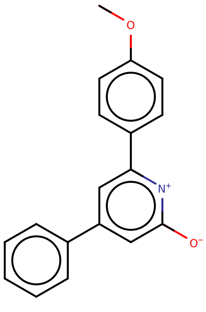
MEMTYR10	MTYROS	NEGBUW	NULNAL

29	30	31	32
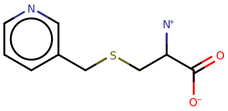	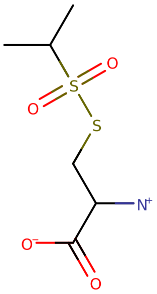	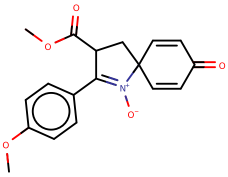	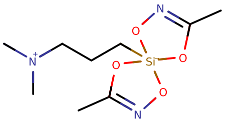
OCAZUO	ODESUO	POSRUM	QAJFOW

33	34	35	36
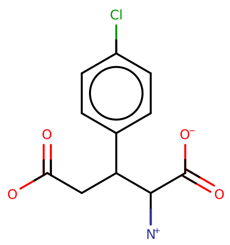	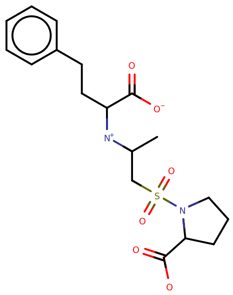	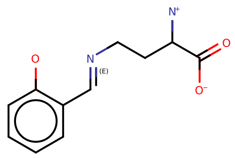	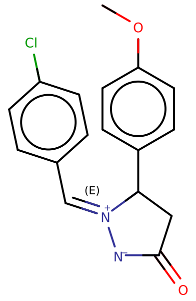
RAYWOD	RUQKAR	SAQQIK	TOJZID
**37**	**38**	**39**	**40**
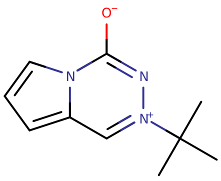	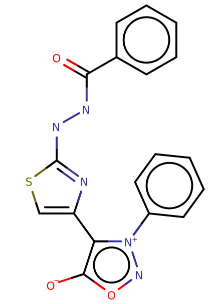	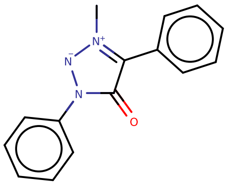	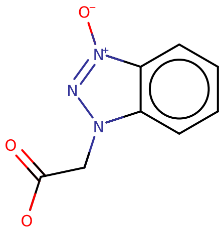
WEPBAU	WOYVUB	XEDGOE	YELKEI
**41**	**42**		
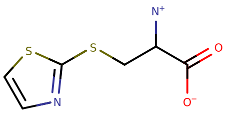	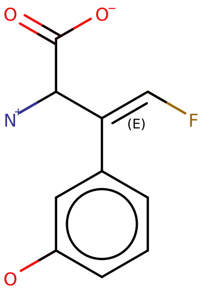		
ZEJPEM	ZULWEI		

J.

The excited states properties of the full database can be found in http://datacat.liverpool.ac.uk/1472/ and optoelectronic properties of materials considered in this study can be extracted from 10.5281/zenodo.6372570.

References.

(1) Nematiaram, T.; Padula, D.; Landi, A.; Troisi, A. On the Largest Possible Mobility of Molecular Semiconductors and How to Achieve It. *Adv. Funct. Mater.* **2020**, *30* (30), 1–10. https://doi.org/10.1002/adfm.202001906.

(2) Yuen, A. P.; Bamsey, N. M.; Hor, A. M.; Preston, J. S.; Klenkler, R. A.; Jovanovic, S. M.; Loutfy,

R. O. Rubrene as an Additive in M-Phthalocyanine/Fullerene Organic Solar Cells. *Sol. Energy Mater. Sol. Cells* **2011**, *95* (11), 3137–3141. https://doi.org/10.1016/j.solmat.2011.04.041.

(3) Huang, J.; Yu, J.; Guan, Z.; Jiang, Y. Improvement in Open Circuit Voltage of Organic Solar Cells by Inserting a Thin Phosphorescent Iridium Complex Layer. *Appl. Phys. Lett.* **2010**, *97* (14), 1–4. https://doi.org/10.1063/1.3492838.

(4) Berrehar, J.; Schott, M.; Delannoy, P. Optical Absorption of Crystalline Tetracene in the Low-Energy Tail of the So→S1 Transition. *Phys. Status Solidi* **1975**, *32* (1), K37–K39. https://doi.org/10.1002/pssa.2210320151.

(5) Moh, A. M.; Khoo, P. L.; Sasaki, K.; Watase, S.; Shinagawa, T.; Izaki, M. Growth and Characteristics of C8-BTBT Layer on C-Sapphire Substrate by Thermal Evaporation. *Phys. status solidi* **2018**, *215* (11), 1700862. https://doi.org/10.1002/pssa.201700862.

(6) Nematiaram, T.; Padula, D.; Troisi, A. Bright Frenkel Excitons in Molecular Crystals: A Survey. *Chem. Mater.* **2021**, *33* (9), 3368–3378. https://doi.org/10.1021/acs.chemmater.1c00645.

(7) Ishii, K.; Kinoshita, M.; Kuroda, H. Dielectric Constant Measurement on Organic Crystalline Powder. *Bull. Chem. Soc. Jpn.* **1973**, *46* (11), 3385–3391. https://doi.org/10.1246/bcsj.46.3385.
